# CRISPR/Cas9-mediated heterozygous knockout of the autism gene CHD8 and characterization of its transcriptional networks in cerebral organoids derived from iPS cells

**DOI:** 10.1186/s13229-017-0124-1

**Published:** 2017-03-20

**Authors:** Ping Wang, Ryan Mokhtari, Erika Pedrosa, Michael Kirschenbaum, Can Bayrak, Deyou Zheng, Herbert M. Lachman

**Affiliations:** 1 Department of Genetics, Albert Einstein College of Medicine, 1300 Morris Park Ave, Bronx, NY USA; 2 Department of Psychiatry and Behavioral Sciences, Erciyes University School of Medicine, Kayseri, Turkey; 3 Erciyes University School of Medicine, Kayseri, Turkey; 4 Department of Neurology, Albert Einstein College of Medicine, 1300 Morris Park Ave, Bronx, NY USA; 5 Department of Neuroscience, Albert Einstein College of Medicine, 1300 Morris Park Ave, Bronx, NY USA; 6 Department of Medicine, Albert Einstein College of Medicine, 1300 Morris Park Ave, Bronx, NY USA

**Keywords:** DLX6-AS1, Distal-less homeobox, Gabaergic, Cancer, Autism, Schizophrenia, Bipolar disorder, TCF4, HMGA2, ZNF132, Wnt, Beta-catenin

## Abstract

**Background:**

*CHD8* (chromodomain helicase DNA-binding protein 8), which codes for a member of the CHD family of ATP-dependent chromatin-remodeling factors, is one of the most commonly mutated genes in autism spectrum disorders (ASD) identified in exome-sequencing studies. Loss of function mutations in the gene have also been found in schizophrenia (SZ) and intellectual disabilities and influence cancer cell proliferation. We previously reported an RNA-seq analysis carried out on neural progenitor cells (NPCs) and monolayer neurons derived from induced pluripotent stem (iPS) cells that were heterozygous for *CHD8* knockout (KO) alleles generated using CRISPR-Cas9 gene editing. A significant number of ASD and SZ candidate genes were among those that were differentially expressed in a comparison of heterozygous KO lines (*CHD8* ^+/−^) vs isogenic controls (*CHD8* ^+/−^), including the SZ and bipolar disorder (BD) candidate gene *TCF4*, which was markedly upregulated in *CHD8* ^+/−^ neuronal cells.

**Methods:**

In the current study, RNA-seq was carried out on *CHD8* ^+/−^ and isogenic control (*CHD8* ^+/+^) cerebral organoids, which are 3-dimensional structures derived from iPS cells that model the developing human telencephalon.

**Results:**

*TCF4* expression was, again, significantly upregulated. Pathway analysis carried out on differentially expressed genes (DEGs) revealed an enrichment of genes involved in neurogenesis, neuronal differentiation, forebrain development, Wnt/β-catenin signaling, and axonal guidance, similar to our previous study on NPCs and monolayer neurons. There was also significant overlap in our *CHD8* ^+/−^ DEGs with those found in a transcriptome analysis carried out by another group using cerebral organoids derived from a family with idiopathic ASD. Remarkably, the top DEG in our respective studies was the non-coding RNA *DLX6-AS1*, which was markedly upregulated in both studies; *DLX6-AS1* regulates the expression of members of the *DLX* (distal-less homeobox) gene family. *DLX1* was also upregulated in both studies. *DLX* genes code for transcription factors that play a key role in GABAergic interneuron differentiation. Significant overlap was also found in a transcriptome study carried out by another group using iPS cell-derived neurons from patients with BD, a condition characterized by dysregulated WNT/β-catenin signaling in a subgroup of affected individuals.

**Conclusions:**

Overall, the findings show that distinct ASD, SZ, and BD candidate genes converge on common molecular targets—an important consideration for developing novel therapeutics in genetically heterogeneous complex traits.

**Electronic supplementary material:**

The online version of this article (doi:10.1186/s13229-017-0124-1) contains supplementary material, which is available to authorized users.

## Background

Chromodomain helicase DNA-binding protein 8 (*CHD8*) has emerged as a top ASD candidate gene from multiple exome-sequencing studies [1–4]. Loss of function mutations in the gene have also been found in schizophrenia (SZ) and intellectual disabilities [4–6]. *CHD8* is a ubiquitously expressed member of the CHD family of ATP-dependent chromatin-remodeling factors that play important roles in chromatin dynamics, transcription, and cell survival [7–11]. Previous studies have shown that *CHD8* protein negatively regulates Wnt signaling by interacting with β-catenin: Wnt/β-catenin signaling plays a critical role in normal brain development and has been implicated in bipolar disorder (BD), SZ, ASD, and cancer [4, 10, 12–23]. The effect of CHD8 on the growth of cancer cells appears to be due, in part, to an interaction with p53 [24]. CHD8 also recruits MLL histone methyltransferase complexes to regulate cell cycle genes [25] and binds to the chromatin insulator CTCF [25, 26]. Recent work also shows that CHD8 and other CHD chromatin remodelers regulate embryonic stem cell transcriptional programs by targeting specific nucleosomes that flank nucleosome-free promoter regions [9].

Based on these observations, we have been studying the effects of CHD8 on human neurons and neural progenitor cells (NPCs) using *CHD8* ^+/−^ lines generated in isogenic-induced pluripotent stem (iPS) cells by CRISPR-Cas9 gene editing [8]. Other investigators have been studying the effect of CHD8 on neuronal cells using RNA interference (RNAi) [27–29]. These studies have focused primarily on analyzing downstream targets of CHD8 in order to identify differentially expressed genes (DEGs). This is a particularly useful strategy for studying ASD and SZ candidate genes that function as regulators of gene expression, in order to find converging pathways that could connect many different genetic risk factors into more manageable common molecular subgroups—an idea that could facilitate drug discovery. ASD and SZ candidate genes that code for gene expression regulators (e.g., transcription factors and chromatin-remodeling complexes) represent, along with genes that code for synaptic proteins, calcium channels, potassium channels, and the HLA (MHC) locus, the major categories of validated candidate genes in these conditions [4, 30–33]. Molecular genetic convergence has previously been demonstrated for some candidate genes. For example, the SZ and BD candidate gene *MIR137* has been found to target other candidates: *CSMD1*, *C10orf26*, *CACNA1C*, and *TCF4* [34]. In addition, clinically distinct disorders can be caused by the same risk genes, suggesting that therapies aimed at specific molecular targets could have a therapeutic effect across diagnostic categories [4, 35].

The molecular studies that have targeted CHD8 certainly support the concept of converging molecular targets and pathways. In shRNA knockdown studies and chromatin immunoprecipitation using NPCs, neural stem cells (NSCs), and SK-N-SH neuroblastoma cells, downregulation of CHD8 predicted a disruption of gene networks involved in neurodevelopment and resulted in altered expression of a significant number of other ASD-risk genes [3, 27–29].

Similarly, in our recently published study, a significant number of previously characterized ASD and SZ candidate genes were found to be differentially expressed in *CHD8* ^*+/−*^ NPCs and neurons, compared with isogenic controls [8], and furthermore, DEGs were found to overlap with the downstream targets of several other SZ and ASD candidate genes that code for transcription factors or chromatin regulators, including *TCF4*, *EHMT1*, and *SATB2* [6, 8, 36–38]. This suggests that CHD8 not only has a direct effect on gene expression but has indirect effects as well. We also found that DEGs were enriched for pathways that affect the extracellular matrix (ECM), cell adhesion, neuron differentiation, neuron projection, synaptic transmission, axonal guidance signaling, and WNT/β-catenin and PTEN signaling. In addition, genes involved in head circumference were found to be differentially expressed. This is notable because loss of function *CHD8* mutations are associated with large head circumference, a finding that has been experimentally validated in a zebrafish model [2, 3].

Our previous study was carried out using NPCs and a monolayer neuronal culture system consisting of a fairly heterogeneous array of neurons expressing forebrain, midbrain, and hindbrain markers. Recently, several neuronal differentiation methods have emerged that are more suitable for SZ and ASD, one of which is the direct conversion of iPS cells into 3-dimensional cerebral organoids, which resemble a first trimester developing telencephalon [39–41]. This is particularly appropriate for studying neurodevelopmental disorders that are associated with cognitive dysfunction. We have also demonstrated that the organoid system is ideal for studying gene × environment interactions relevant to neuropsychiatric and neurodevelopmental disorders [39].

The few studies that have been carried out so far using cerebral organoids as a model system have been revealing. Mariani et al., for example, showed that genes involved in cell proliferation, neuronal differentiation, synaptic assembly, and GABAergic inhibitory neuron development were differentially expressed in an idiopathic ASD family [42]. And, using a somewhat different organoid differentiation protocol, Lancaster et al. showed that cerebral organoids derived from patients with *CDK5RAP2* loss of function variants and microcephaly have premature neuronal differentiation [41].

Accordingly, we have expanded our transcriptome analysis of CHD8 target genes in cerebral organoids derived from *CHD8* ^*+/−*^ iPS cells and isogenic controls. The DEGs reported here validate many of the findings in our previous analysis in NPCs and monolayer neuronal cultures. In particular, we show that *CHD8* haploinsufficiency again leads to a substantial increase in *TCF4* expression [8]. In addition, significant overlap was found with the DEGs previously identified in the Mariani et al. study, which was carried out using subjects with idiopathic ASD in whom the responsible genetic variant could not be unequivocally characterized [42]. The long non-coding antisense RNA *DLX6-AS1*, a regulator of GABAergic interneuron development [43], was the top DEG in both. Considering the genetic heterogeneity found in ASD and SZ, the molecular convergence on *DLX6-AS1* between *CHD8* and an uncharacterized ASD-causing genetic variant is striking.

## Methods

### Development of iPSCs from skin fibroblasts

We have been developing iPS cells from controls and patients with 22q11.2 del diagnosed with SZ or schizoaffective disorder [44]. One of the male control samples was used to generate the CHD8^+/−^ lines. The control was recruited from the Albert Einstein College of Medicine (AECOM). The study and consent forms were approved by the AECOM Institutional Review Board (IRB). Consent was obtained by a skilled member of the research team who had received prior human subjects training. iPSC lines were generated from fibroblasts obtained from skin biopsies performed by board-certified physicians. The procedure for growing fibroblasts in preparation for reprogramming into iPS cells is detailed in Additional file 1: Supplemental methods.

### Generating *CHD8* KO lines

*CHD8* ^+/−^ lines were developed by introducing a CRISPR-Cas9 vector containing *CHD8* guide sequences into iPS cells by nucleofection [8]. The procedure is described in detail in Additional file 1: Supplemental methods.

### Cerebral organoid differentiation

The protocol is adapted from Mariani et al. [40]. Briefly, iPS cell colonies were maintained on matrigel in mTesr1. To induce cerebral organoid differentiation, iPS cells were pretreated with 50 μM Y27632 in mTesr1 for 1 h at 37 °C. Wells were rinsed with DMEM/F12, and iPS cell colonies were dissociated with accutase for 10 min at 37 °C. Cells were rinsed with DMEM/F12 and collected and counted for aggregate formation. Following the Stem Cell Technologies protocol, 3.0 × 10^6^ cells were used to create 10,000 cell aggregates using an AggreWell™ plate. For the first 6 days, aggregates were cultured in mTesr1 supplemented with 500 ng/ml DKK-1, 1.5 μg/ml BMPRIA-Fc, and 10 μM SB431542. On day 6, aggregates were removed from the AggreWell™ plate, according to the Stem Cell Technology protocol, and transferred to a 24-well ultra-low attachment plate. On day 18, 1% N2 supplement was added to the medium. On day 25, aggregates were plated onto a 4-well chamber slide coated with 10 μg/ml polyornithine, 2.5 μg/ml laminin, and 50 μg/ml fibronectin, and cultured in Neurobasal medium supplemented with 2% B27 and 2 mM l-glutamine until day 50. Organoids were detached, and RNA was extracted. Organoids are composed of a mixture of GABAergic and glutamatergic neurons, and radial glia progenitor cells, and have gene expression profiles that resemble a first trimester telencephalon (Additional file 2: Figure S1) [39, 40, 45]. For immunohistochemistry (IHC), samples were fixed with 4% paraformaldehyde and 25% sucrose, and then embedded in O.C.T (optimal cutting temperature) (see Additional file 1: Supplemental methods for IHS methodology).

### RNA-seq

Total RNA was isolated using the miRNeasy kit (Qiagen) according to the manufacturer’s instructions. We obtained 101 bp paired-end RNA-seq reads from an Illumina HiSeq 2500 instrument. Adapters and low quality bases in reads were trimmed by trim_galore (http://www.bioinformatics.babraham.ac.uk/projects/trim_galore/). We employed Kallisto (v0.42.5) [46] to determine the read count for each transcript and quantified transcript abundance as transcripts per kilobase per million reads mapped (TPM), using gene annotation in the GENCODE database (v18) [47]. Then we summed the read counts and TPM of all alternative splicing transcripts of a gene to obtain gene expression levels. We restricted our analysis to 12,898 expressed genes with an average TPM >1 in either wild type or *CHD8* ^+/−^ samples. DESeq2 [48] was used to identify DEGs (false discovery rate (FDR) <0.05). The software DAVID (v6.8 Beta) [49, 50] was used for Gene Ontology (GO) analysis, with the 12,898 expressed genes as background. Ingenuity pathway analysis (IPA) (https://www.qiagenbioinformatics.com/) was used for canonical pathway analysis, using the ingenuity knowledge base (genes only) as background. The RNA-seq data have been deposited in Gene Expression Omnibus (GEO: accession number GSE85417).

### Quantitative real-time PCR (qPCR)

qPCR was carried out on reverse transcribed PCR using the 2^−ΔΔCt^ method as previously described [51, 52]. A detailed description and the primers used for this analysis can be found Additional file 1: Supplemental methods.

### ASD/SZ-risk gene sets

For ASD, we compared our DEG list with the following ASD gene sets: SFARI [https://gene.sfari.org/autdb/GS_Home.do] (genes scored as high confidence, to minimal evidence and syndromic); AutismKB (core dataset) [53]; a set of high-confidence ASD genes (Willsey_ASD) [54]; genes predicted by whole exome sequencing and co-expression network analysis (Liu_ASD) [55]; candidate genes with de novo mutations from massive whole exome sequencing (Iossifov_ASD) [56]; and candidates from the same dataset focusing on a combination of de novo and inherited mutations resulting in a high-confidence list (FDR < 0.1) (DeRubeis_ASD) [57]. The two SZ gene lists were from the SZ gene database [58] and a recent genome-wide association study (GWAS) report (SZC GWAS) [33]. These gene lists can be obtained from our previous publication [8].

### Comparison of *CHD8*^+/−^ DEGs with idiopathic ASD organoids

The DEG list from *CHD8* ^*+/−*^ organoids was compared to the DEG lists generated from idiopathic autism patient-specific organoids described by Mariani et al. [42]. The latter were obtained from two developmental stages, after 11 and 31 days of terminal differentiation (TD11 and TD31).

### Comparison of *CHD8*^*+/−*^ DEGs with BD patient-derived neurons

DEG lists from Mertens et al. [59] were derived from the file “GSE58933_Jun_All_Data.txt.gz” in the GEO “GSE58933” record. For a comparison with our *CHD8* KO samples, we applied the same criteria used in the original study for identifying DEGs (log2 (fold change) ≥1 and *p* ≤ 0.05).

### Statistics

To determine if DEGs overlapped with or were significantly enriched with a specific gene set, 12,893 expressed genes in our samples were used as background for Fisher’s exact test. Statistics tests were conducted in R (http://www.R-project.org/). Common genes between two gene lists were input to DAVID (beta 6.8) for GO term analysis.

## Results

RNA-seq was carried out on cerebral organoids derived from *CHD8* KO iPS cells; two isogenic controls (*CHD8* ^+/+^) and four heterozygotes (*CHD8* ^+/−^). The *CHD8* ^+/−^ samples contain a *CHD8* KO allele with either a 10-base pair deletion (clones A, B, and C) or a 2-base pair deletion (D), both of which lead to frameshift mutations and premature stop signals in exon 1 [8]. The KO lines were derived from *CHD8* ^+/+^A; the other control, *CHD8* ^+/+^B, was a different iPS cell clone from the same subject. We previously showed that heterozygous KO leads to a ~50% reduction in CHD8 protein [8]. Similarly, quantitative immunohistochemistry showed a 54% decrease in CHD8 immunoreactivity in *CHD8* ^+/−^ compared with *CHD8* ^+/−^ organoids (analyzed in 15 random fields, *p* = 7.2E-13) (Additional file 2: Figure S1).

**Fig. 1:**
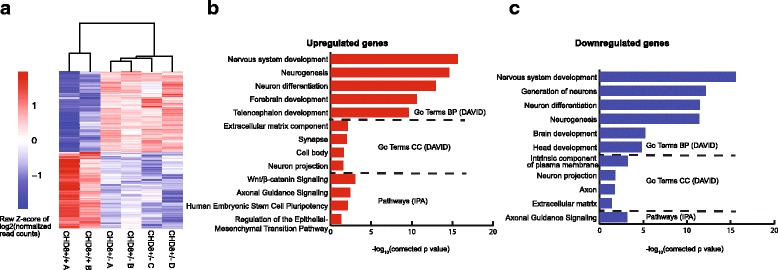
Heat map and summary of GO terms and pathways. **a** The heat map shows differentially expressed genes between controls (*CHD8* ^+/+^) and heterozygous knockouts (*CHD8* ^+/−^). Enriched GO terms by DAVID (*top*) and pathways by IPA (*bottom*) for upregulated (**b**) and downregulated (**c**) genes in *CHD8* ^+/−^ organoids. *P* values were corrected by the Benjamini method [147]

The RNA-seq data quality is shown in Additional file 3: Table S1. A total of 12,893 expressed genes were detected, and DESeq2 was used to identify DEGs, as described in detail in the “Methods” section. Using a cutoff of FDR < 0.05, there were 559 DEGs when the *CHD8* ^+/+^ organoids were compared with *CHD8* ^+/−^; 288 genes increased in the KO, 271 decreased. The DEGs separated our sample into two groups, as seen in the heat map shown in Fig. 1a. The entire list of DEGs is in Additional file 4: Table S2. CHD8 mRNA itself was not significantly differentially expressed based on our RNA-seq analysis. The KO allele, however, showed a much lower level of expression than the WT allele in the organoids (Additional file 1), probably due to nonsense mediated decay. Overall, though, the decrease in CHD8 mRNA was not proportional to the decrease in CHD8 protein, similar to our observations in NPCs [8]. The relatively imprecise correlation between mRNA and protein levels is found for many genes and can be due to a number of factors [60]. However, the mechanism of the discrepancy between CHD8 mRNA and protein is not known and will require further investigation.

We should also point out that among the three *CHD8* alternatively spliced transcripts in the GENCODE annotation, the two containing the exon 1 accounted for 70 ~ 80% of the CHD8 transcripts in the WT organoids and 60–70% in the *CHD8* ^+/−^, based on our RNA-seq data (Additional file 1).

Of the 559 DEGs, 203 have CHD8 binding sites in their promoters, using data from a ChIP-seq study carried out on NPCs by Sugathan et al. (see Additional file 4: Table S2, column I) [28]. The finding that such a large fraction of DEGs are direct targets of CHD8 confirms the validity of our RNA-seq findings. However, it also shows that many downstream genes are indirect targets of CHD8, most likely through the actions of other genes coding for transcription factors and chromatin-remodeling proteins that are directly affected by CHD8, such as *TCF4*, *POU3F2*, *SMARCA4*, *SOX2*, and *PAX6.* This result is consistent with our previous report [8].

The software DAVID was used to identify enriched GO pathways in DEGs using 12,893 expressed genes as background [49]. IPA was used for canonical pathways and disease association. The top GO terms (Biological Process, (BP)) for genes that were upregulated in the *CHD8* KO organoids were nervous system development, neurogenesis, neuron differentiation ,and forebrain development; the top GO:BP terms for downregulated genes were nervous system development, generation of neurons, and neuron differentiation (Fig.1b, c; Additional file 5: Table S3). Genes coding for components of the ECM were the top cellular component (CC) GO terms for upregulated DEGs, and among the top eight for downregulated DEGs, similar to our previous findings using monolayer neurons [8]. The top enriched IPA canonical pathways were Wnt/β-catenin signaling and axonal guidance for upregulated genes and axonal guidance for downregulated genes. An enrichment of DEGs involved in Wnt/β-catenin signaling is similar to that found in our previous transcriptome analysis on *CHD8* ^*+/−*^ NPCs and neurons [8], as well as findings by other investigators [10, 20, 24], firmly establishing that altered expression of *CHD8* disrupts this critical signaling pathway.

As a complementary analysis, we also applied TopHat and DESeq2 for aligning the RNA-seq reads and for DEG analysis, respectively, as we previously carried out [8]. This resulted in 811 DEGs (Additional file 4: Table S2 sheet 2), 534 of which were included in the DEG list from Kallisto/DESeq2 analysis. GO analysis showed an enrichment of similar GO terms in the two DEG lists, with “neuron system development” being the top term for both upregulated and downregulated genes (Fig. 1).

Overall, the findings show that *CHD8* directly, or indirectly through effects on other transcription factors and chromatin regulators, regulates a program of gene expression that affects critical aspects of brain development (e.g., neurogenesis, neuron differentiation, and axonal guidance).

### Comparison between organoid data and NPCs and monolayer neurons

**Fig. 2:**
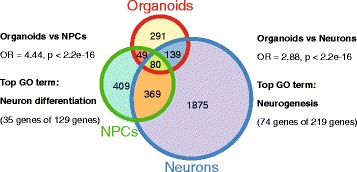
Overlapping DEGs in organoids compared with NPCs and monolayer neurons from previous study [8]

We compared current transcriptome data with our previous study using NPCs and monolayer neurons [8]. There is a significant overlap between the studies, with nearly 50% of DEGs in organoids showing differential expression in NPCs and neurons (neurons odds ratio [OR] = 2.88, *p* < 2.2E-16; NPCs OR = 4.44, *p* < 2.2E-16, Fisher’s test) (Fig. 2; see Additional file 4: Table S2 for overlapping genes). The top GO terms for overlapping genes were neuron differentiation and neurogenesis, respectively, which is consistent with the main pathway findings in organoids described above.

### qPCR validation

**Fig. 3:**
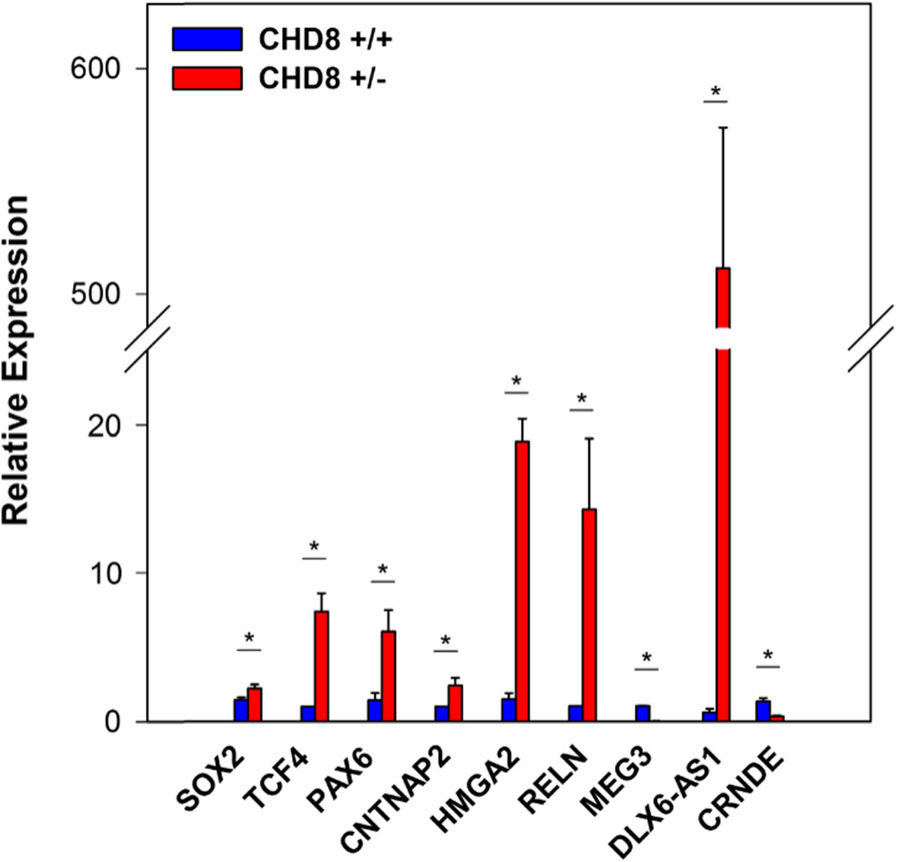
Validation of selected DEGs by qPCR. The RNA samples used in the RNA-seq were used for this analysis. Samples were analyzed in triplicate as described in “Methods” section and Additional file 1: Supplemental methods. Significant differences between control and KO are denoted by an *asterisk* (*). The *p* values derived by Student’s *t* test were as follows: *SOX2*, 0.047; *TCF4*, 0.004; *HMGA2*, 0.00005; *PAX6*, 0.02; *RELN*, 0.02; *CNTNAP2*, 0.04; *DLX6-AS1*, 0.0001; *MEG3*, 0.00002

**Fig. 4:**
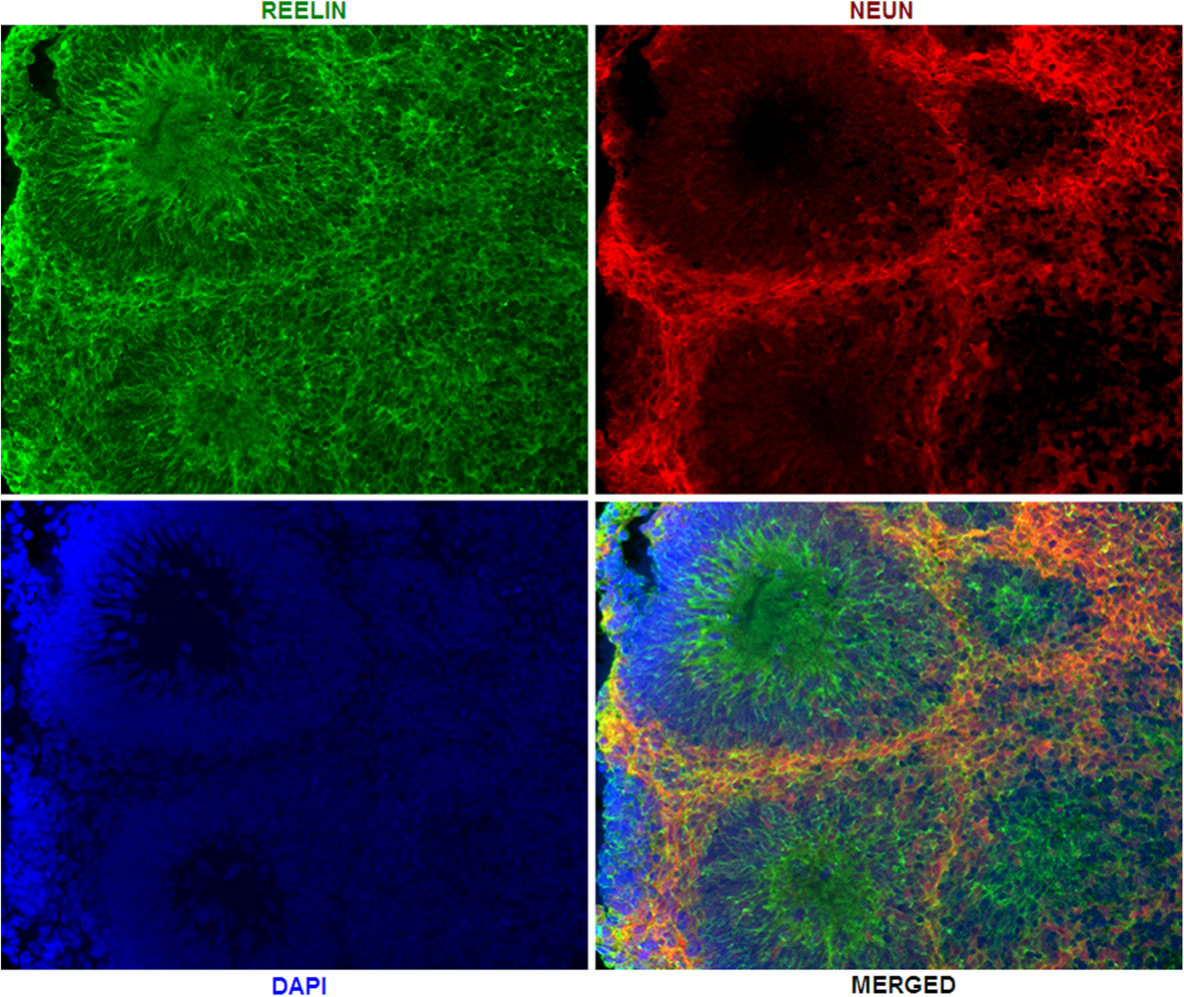
Immunohistochemistry. RELN and the neuronal marker NeuN were visualized as described in Additional file 1: Supplemental methods. The DAPI+ tubular structures are zones of proliferating radial glia progenitors. NeuN+ cells are fields of neurons surrounding the radial glia progenitors

We validated several RNAs of interest by qPCR using the 2^−ΔΔCt^ relative expression method: *SOX2*, *PAX6*, *TCF4*, *CNTNAP2*, *HMGA2*, *RELN*, *MEG3*, *DLX6-AS1*, and *CRNDE* (Fig. 3). These genes were chosen because of their known importance in brain development and disease. *SOX2* and *PAX6* code for transcription factors that influence neural stem cell growth and brain development [60, 61]. *TCF4* codes for a transcription factor; both common and rare variants have been implicated in the etiology of SZ, BD, ASD, and developmental delay [34, 36, 37, 62]. *CNTNAP2* codes for a member of the neurexin family of presynaptic proteins; it too has been implicated in the pathogenesis of SZ and ASD [63–67]. *HMGA2* codes for a non-histone DNA-binding protein that has been implicated in regulating brain growth and head circumference; increased expression was found in our previous transcriptome analysis [8, 68]. *RELN* codes for reelin, a key secreted ECM protein involved in neuronal migration during brain development [69–72]. Altered expression has been found in SZ and ASD [72–77]. As seen in Fig. 4, reelin is expressed throughout the organoids, in fields of neurons as well as in the zone of proliferating radial glia progenitors found in these structures [39, 40, 42]. *MEG3* is a maternally expressed imprinted gene that acts as a tumor suppressor gene in a number of malignancies [21, 78–82]. *DLX6-AS1* and *CRNDE* will be discussed below. qPCR analysis validated the RNA-seq findings for each of these genes.

### Top DEGs and overlap with organoid transcriptome in idiopathic ASD

Two of the top three DEGs in *CHD8* ^+/−^ cerebral organoids were *DLX6-AS1* and *DLX1*, which increased ~39 and 13-fold respectively (Additional file 4: Table S2). They were hardly expressed in controls. *DLX6-AS1* (also known as *EVF2*) forms a complex with DLX1 and DLX2 proteins that subsequently regulates GABAergic interneuron development by increasing *DLX5* and *DLX6* gene expression [43, 83–86]. Strikingly, in a study by Mariani et al., *DLX6-AS1* was also the top DEG in a transcriptome analysis carried on day 11 cerebral organoids derived from members of a family with idiopathic ASD, and the 6th top DEG in day 31 organoids [42]. *DLX1* was differentially expressed at both time points as well.

In addition to *DLX6-AS1*, among the top 15 DEGs in *CHD8* ^+/−^ cerebral organoids, 11 were also DEGs in the Mariani et al. study in either or both of their day 11 and day 31 organoids, and the direction of change was the same (Table 1). This included genes involved in brain development, several of which have been implicated in ASD, including *FZD8*, *PAX6*, *SLC1A3*, *EOMES* (*TBR2*), and *MPPED1*.

**Table 1:** Top 15 DEGs in CHD8^+/−^ vs idiopathic ASD cerebral organoids

CHD8^+/−^	Log2FC	Padj	Log2FC_11	FDR	Log2FC_31	FDR
DLX6-AS1	5.34	3.85E-86	4.72	1.70E-75	4.61	7.03E-37
ARMCX1	−2.48	4.47E-34	NS	NS	NS	NS
DLX1	3.84	5.97E-32	2.14	1.38E-11	1.35	8.69E-03
FZD8	2.21	1.17E-26	1.19	2.35E-04	NS	NS
CPNE6	3.55	1.47E-24	NS	NS	NS	NS
PAX6	2.03	2.05E-22	NS	NS	1.29	2.23E-04
SLC1A3	1.61	2.05E-22	1.13	9.21E-06	NS	NS
EOMES	3.10	9.04E-22	2.96	1.85E-17	2.79	1.17E-06
MPPED1	2.92	3.31E-21	1.57	4.50E-05	1.41	7.29E-03
COL25A1	2.67	4.42E-21	NS	NS	NS	NS
SCGN	3.22	9.46E-20	2.15	1.25E-06	1.37	1.57E-02
STK17B	2.08	9.46E-20	NS	NS	NS	NS
LIX1	2.14	8.70E-19	NS	NS	0.83	2.53E-02
BCL11B	1.63	7.21E-18	0.95	4.85E-02	2.16	2.36E-12
SHISA2	2.14	2.29E-17	1.41	1.58E-06	NS	NS

Top 15 DEGs in *CHD8* ^+/−^ are shown in left columns with fold change (FC) and *p* value adjusted for genome-wide significance (padj). The right columns show the FC and false discovery rate (FDR) values for the same genes, derived from the Mariani et al. study [42]. Log2FC_11 is from day 11 cerebral organoids, while log2FC_31 is from day 31 organoids. Note that DLX6-AS1 was the top DEG in day 11 organoids and the 6th top DEG in day 31 organoids

**Fig. 5:**
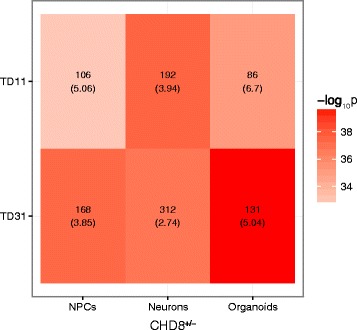
Overlap in DEGs between *CHD8* and idiopathic ASD organoids. *CHD8* KO DEGs were compared with DEGs from day 11 and day 31 organoids (TD11, TD31) derived from individuals with idiopathic ASD [42]. The *number* in each panel shows the number of overlapping genes, which can be seen in Additional file 5: Table S4. The *numbers in parentheses* are the odds ratios. *Color* represents *p* value from Fisher’s test

Aside from the top DEGs, overall, there was a significant overlap in DEGs between our *CHD8* ^+/−^ organoids and idiopathic ASD day 11 and day 31 organoids in the Mariani et al. study (Fig. 5; Additional file 6: Table S4). Similarly, significant overlap was detected in a comparison of DEGs from our previous study on NPCs and monolayer neurons with idiopathic ASD organoids. The most significant overlap was found in the comparison between *CHD8* ^*+/−*^ organoids and day 31 organoids from the Mariani et al. study, which showed that 23% of DEGs were the same (131/560; OR = 5.04; *p* = 1.34E-40). The top GO terms for overlapping genes were nervous system development, neuron differentiation, and neurogenesis for day 11 and day 31 organoids (Additional file 6: Table S4).

Although the degree of overlap is impressive, a key difference in our respective studies is that *FOXG1* was a top, upregulated DEG in the idiopathic ASD organoids, but not in the *CHD8* ^*+/−*^ samples; reducing *FOXG1* by RNAi rescued the over-abundance of GABAergic interneurons found in the idiopathic ASD organoids [42]. Conversely, *TCF4,* a major DEG in all of our *CHD8* ^*+/−*^ samples, was not differentially expressed in the idiopathic ASD organoids. This suggests that unique expression changes can occur in key genes despite the extensive overlap in transcriptomes, which could conceivably limit the full therapeutic impact of novel drugs that target common pathways.

### *CHD8* haploinsufficiency and WNT-β-catenin signaling

**Fig. 6:**
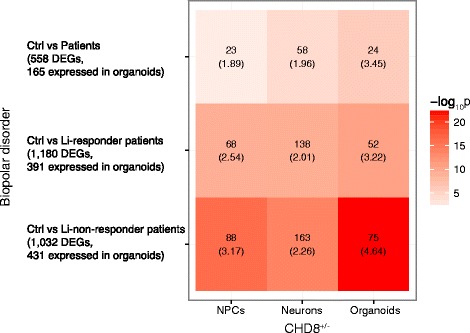
Overlap in DEGs between *CHD8* KO and BD neuronal cells. *CHD8* KO DEGs from the current study (organoids) and our previous study (NPCs, neurons) [42] were compared with DEGs found in neurons derived from lithium responder and lithium non-responder patients with BD, as described in “Methods” section. The *number* in each panel shows the number of overlapping genes, which can be seen in Additional file 6: Table S5. The *numbers in parentheses* are the odds ratios

WNT/β-catenin signaling is a key pathway in the developing brain that is dysregulated in neuropsychiatric disorders, as well as in various cancers [4, 10, 12–23]. Thus, the finding that *CHD8* binds to β-catenin, inhibiting its transcriptional effects, [10, 20] is relevant to the role of *CHD8* in both neuropsychiatric and neurodevelopmental disorders, as well as cancers. Recently, however, CHD8 was shown to be a positive regulator of WNT/β-catenin signaling in human NPCs [87]. Among the neuropsychiatric disorders, WNT/β-catenin is particularly relevant to BD because lithium salts, which are used to treat the condition, inhibit GSK3β, which would be expected to result in an increase in β-catenin levels (constitutive GSK3β activity leads to β-catenin degradation) [88–92]. With these considerations in mind, as well as our finding that WNT/β-catenin signaling is the top pathway for upregulated DEGs in CHD8^+/−^ organoids (Fig. 1b, c), we compared our DEG list with those found in a recent study by Mertens et al. in which transcriptome analyses were carried out in iPS cell-derived neurons from BD patients who were clinically responsive or not responsive to lithium [59]. In addition, we also evaluated the DEG list derived from NPCs and monolayer neurons from our previous study, which also showed that WNT/β-catenin was a top pathway among DEGs in monolayer neurons [8]. Using the same criteria for defining DEGs in the Mertens et al. study (log2 fold change ≥1 and *p* ≤ 0.05), significant overlap was found in each comparison (Fig. 6; Additional file 7: Table S5). Note that only 30–50% of DEGs in the Mertens et al. study were expressed in our organoids, which is probably due to differences in the differentiation protocols used. However, the most significant overlap (*p* ≤ 5.73E-23; OR = 4.64) occurred in the comparison between our *CHD8* ^+/−^ DEGs and the lithium non-responders in Mertens et al. (Interestingly, *DLX6-AS1* and *DLX1* were in this group of overlapping DEGs; Additional file 7: Table S5).

The top GO terms for overlapping genes were similar for both the Li non-responder and Li responder vs CHD8^+/−^ organoid DEGs; nervous system development, neuron differentiation, and neurogenesis (Additional file 7: Table S5). However, one GO term found exclusively in the former was axonogenesis. Although lithium is extremely useful in a substantial proportion of BD patients, it is also used on occasion to treat patients diagnosed with SZ and ASD, especially as adjunctive therapy for those with a mood component and for refractory patients to augment the effect of anti-psychotic medications [88, 93–98]. Although there was a greater association to the lithium non-responder group, considering that there is significant overlap with the lithium-responder group as well could have therapeutic implications.

### ASD and SZ candidates in *CHD8*^*+/−*^ DEGs

**Fig. 7:**
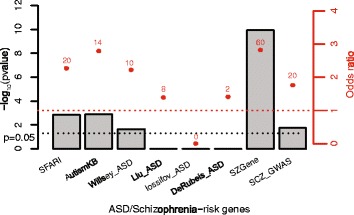
Overlap between *CHD8* KO DEGs and ASD and SZ candidate genes. *CHD8* KO DEGs were evaluated for enrichment of ASD and SZ candidate genes. *Bars* represent *p* value (Fisher’s exact test, one-tailed), while *red dots* represent odds ratio of overlap. The *number above each red dot* shows the number of overlapping genes, which can be found in Additional file 7: Table S6

The protein-coding DEGs were considered for their over-representation of SZ and ASD candidate genes using a variety of sources, as described in the “Methods” section and our previous study [8]. As seen in Fig 7, among the *CHD8* ^+/+^ vs *CHD8* ^+/−^ DEGs, there was significant enrichment of ASD candidate genes in the SFARI, AutismKB, and Willsey ASD datasets, and an even more significant enrichment of SZ candidates in the SZGene and SZ GWAS lists (see Additional file 8: Table S6 for complete list). Enriched GO terms were identified for the overlapping genes in three of these datasets: SFARI, AutismKB, and SZ GWAS. The top GO terms for SFARI and AutismKB were similar; forebrain development, telencephalon development, and pallium development were the most significant (Additional file 8: Table S6). For the SZ GWAS data set, the top GO terms for overlapping genes were somatodendritic compartment, synapse, neuron projection, cell body, and axon. The differences between the ASD and SZ sets of overlapping genes reflect the observation that *CHD8* haploinsufficiency is a risk factor for both groups of conditions and suggest that disruption of different molecular pathways is involved in the increased risk and differences in clinical presentation.

### DEGs involved in head circumference/brain volume

Patients with loss of function *CHD8* mutations typically have large head circumferences, a finding confirmed in a zebrafish model [2, 3]. In our previous study, 7 out of 12 genes (*TESC*, *DDR2*, *HMGA2*, *SBNO1*, *FAT3*, *BCL2L1*, and *MSRB3*) implicated in brain size in genome-wide association studies were differentially expressed in *CHD8* ^+/−^ NPCs and neurons [8, 68, 99, 100]. However, among these genes, only *HMGA2* (high mobility group AT-hook 2) expression was similarly affected in *CHD8* KO organoids. In addition, one gene, *DCC* (deleted in colorectal carcinoma), which was not differentially expressed in the previous study, was significantly decreased in *CHD8* ^+/−^ organoids. Both the *HMGA2* and *DCC* gene loci have CHD8 binding sites [28]. The findings show unequivocally that *HMGA2* expression is regulated by CHD8.

### ncRNAs

In addition to *DLX6-AS*, there were 19 other non-coding RNAs (ncRNAs) that were differentially expressed in *CHD8* ^+/−^ organoids (Table 2), of which 9 are CHD8 targets based on published ChIP findings [28]. Several of the differentially expressed ncRNAs we detected have been implicated in neuropsychiatric disorders. One is the *RMST* locus, which contains a microRNA involved in forebrain development through its modulation of WNT/β-catenin signaling and has been found to regulate neurogenesis through an interaction with SOX2 [13, 101]. Another is *MIAT* (also known as *GONAFU*); RNA levels were found to be expressed at higher levels in parvalbumin GABAergic interneurons in SZ subjects [102] and have been found to regulate fear-related anxiety traits in mice [103]. *MIAT* also binds to several splicing factors and may affect splicing of the SZ-associated *ERBB4* and *DISC1* genes in response to neuronal activation [104]. Finally, *CRNDE* expression is significantly elevated in iPS cell-derived neurons from patients with SZ who have 22q11.2 deletions [105]. Although the molecular effects of most ncRNAs have not been determined, many function at the gene expression level through their regulation of chromatin architecture and nuclear organization, which, if disrupted, could potentially alter neurodevelopmental molecular programs [43, 82, 104, 106–111].

**Table 2:** Differentially expressed ncRNAs

Gene_name	Gene_type	NPC_binding	Log2FC	Padj
DLX6-AS1	antisense	0	5.34E + 00	3.85E-86
EMX2OS	antisense	0	1.10E + 00	3.79E-02
LINC00340	lincRNA	1	−7.78E-01	1.54E-04
TERC	lincRNA	0	−1.07E + 00	1.18E-03
CRNDE	lincRNA	1	−9.63E-01	1.39E-02
MEG3	lincRNA	1	−1.24E + 00	1.39E-02
RMST	processed_transcript	0	−1.23E + 00	6.41E-07
MIAT	processed_transcript	1	−1.28E + 00	6.10E-06
NEFL	processed_transcript	0	−1.18E + 00	2.08E-04
TMEM191A	processed_transcript	0	−7.74E-01	1.33E-02
SPPL2B	processed_transcript	1	−6.41E-01	2.32E-02
SCARNA22	sense_intronic	0	−1.31E + 00	1.56E-03
SNHG3	sense_intronic	1	−7.94E-01	1.41E-02
SOX2-OT	sense_overlapping	1	9.29E-01	9.81E-04
SNORA31	snoRNA	0	−1.03E + 00	2.41E-02
SNORA7B	snoRNA	1	−1.03E + 00	3.30E-02
SCARNA13	snoRNA	0	−7.08E-01	3.77E-02
SNORA73B	snoRNA	1	−7.89E-01	4.61E-02
RNU2-59P	snRNA	0	−9.94E-01	1.76E-02
RNU6-15P	snRNA	0	−1.07E + 00	4.99E-02

FC is fold change: *CHD8* ^+/−^/*CHD8* ^++^. Padj is adjusted *p* value. CHD8 binding based on ChIP-seq in NPCs by Sugathan et al. [42]

## Discussion

One of the most interesting characteristics of *CHD8* is its diverse effect on several neuropsychiatric and neurodevelopmental disorders and cancer. Although ASD and cancer differ fundamentally in a key aspect regarding loss of function *CHD8* mutations and disease in that the former are due to germline mutations, while the latter are usually somatic, it is not surprising that a chromatin and transcriptional regulator like CHD8 would play a role in both types of conditions. Indeed, in recently published pathway network analyses and sequencing studies, overlap was found for several ASD candidate genes and cancer [30, 112]. Correspondingly, germline mutations in *NF1,* which cause neurofibromatosis type I, often display autistic-like behaviors [113, 114]. The molecular genetic overlap suggests that some novel cancer therapies currently being developed, especially those that target the epigenome might be beneficial in treating subgroups of individuals with neurodevelopmental disorders [112, 115].

The mechanisms by which loss of function *CHD8* mutations increase cancer risk are likely to be multifactorial. Part of the effect appears to be due to the direct interaction between CHD8 protein with β-catenin and p53, and an effect on the cell cycle [2, 24, 25]. An effect mediated by β-catenin is given additional support by our transcriptome analysis. In addition, based on our findings, CHD8 may also contribute to malignant transformation indirectly through its effects on other genes, which were found to be differentially expressed in this study and have been implicated in malignant transformation, such as *SMARCA4*, *POU4F1*, *ARMCX1*, *HMAG2*, *DCC*, and *ZNF132* (Additional file 4: Table S2) [116, 117]. Several of the differentially expressed ncRNAs we show on Table 2 have also been found to be associated with cancer development, including *TERC*, which codes for the RNA component of telomerase; telomere shortening is a feature of malignant transformation and aging [118]. *MEG3*, as noted above, *CRNDE*, *LINC00340*, *and RMST* (rhabdomyosarcoma 2 associated transcript) is also found in various cancers [118–124]. These findings suggest that the effect of CHD8 on malignant transformation is multifactorial and not simply due to a direct interaction with the Wnt/β-catenin signaling pathway.

Similarly, the role of *CHD8* on neuropsychiatric and neurodevelopmental disorders is multifactorial, with both direct effects on downstream targets, such as β-catenin, and indirect effects mediated by dysregulated expression of other transcription factors and chromatin remodelers. The best example of this is the SZ and BD candidate gene *TCF4*, which codes for a basic helix-loop-helix transcription factor [125]. *CHD8* haploinsufficiency leads to a ~2-fold increase in *TCF4* expression in cerebral organoids and NPCs and neurons (Additional file 4: Table S2) [8]. In addition, pathway analysis showed extensive overlap with TCF4 targets, and CHD8 binds to the *TCF4* gene locus [8, 28]. An increase in *TCF4* expression has also been found in iPS cell neurons and fibroblasts derived from SZ patients [126, 127]. In addition, *TCF4* is upregulated by loss of function mutations in the SZ candidate *MIR137*, and overexpression in the forebrain of mice leads to cognitive impairments and deficits in pre-pulse inhibition [128–130]. Overall, the findings strongly suggest that TCF4 and CHD8 cooperate to influence neuronal differentiation and brain development, and that *TCF4* overexpression is a key feature in SZ and BD.

On the other hand, loss of function *TCF4* mutations have been found in patients with Rett syndrome-like phenotypes [131, 132], ASD [133] and Pitt–Hopkins syndrome, which is characterized by ASD, intellectual disabilities, and microcephaly [134, 135]. Thus, *TCF4* gene dosage in either direction adversely affects brain development.

Other transcription factors and chromatin regulators that are significantly affected by *CHD8* haploinsufficiency that are also ASD and SZ candidate genes include *POU3F2* and *AUTS2. POU3F2* expression is significantly decreased in *CHD8* ^+/−^ in cerebral organoids, as well as in NPCs and neurons, while *AUTS2* expression is significantly decreased in *CHD8* ^+/−^ in cerebral organoids and neurons, but not NPCs (Additional file 4: Table S2) [8]. *POU3F2* codes for a member of the POU family of transcription factors, and has been implicated in SZ, and more recently in BD, developmental delay, and intellectual disability [136, 137]. Its critical role in neuronal differentiation is highlighted by the finding that it is one of the three factors, along with MYT1L and ASCL1, used in the direct reprogramming of fibroblasts into neurons [138]. *AUTS2* codes for a chromatin-remodeling protein that functions as a component of the polycomb repressive complex 1 (PRC1); loss of function mutations can lead to ASD, SZ and intellectual disabilities [139–141]. Also, it is interesting to note, in the context of the ASD/cancer connection, that *AUTS2* is part of a translocation commonly found in childhood B cell precursor acute lymphoblastic leukemia [142].

One of the most interesting downstream targets of CHD8 we found regarding neurodevelopmental and neuropsychiatric disorders is the non-coding RNA, *DLX6-AS1*, which was the top DEG in our *CHD8* ^+/−^ organoids, as well as organoids derived from a family with idiopathic ASD [42]. *DLX6-AS1* overlaps with *DLX6* and is expressed in the opposite transcriptional orientation. In mice, a splice variant of Dlx6-as1 called Dlx6-as2 (evf2) cooperates with Dlx2 to increase the transcriptional activating function of the Dlx5/6 enhancer [143].

These findings suggest that CHD8 affects GABAergic interneuron development, by modulating *DLX* gene expression. Consistent with this idea is the finding that several genes, in addition to *DLX1*, involved in cerebral cortex GABAergic interneuron differentiation, *FEZF2*, *ARX*, and *CNTN2*, were differentially expressed in *CHD8* ^*+/−*^ organoids (Additional file 4: Table S2). Statistically, the enrichment of genes involved in cerebral cortex GABAergic interneuron differentiation only achieved a trend toward significance (*p* = 3.6E-3; padj = 5.8E-2), which could be due to the relatively small sample size. Abnormalities in cortical GABA interneuron function, in particular, parvalbumin positive and somatostatin positive interneurons have been found in SZ and ASD [144–146].

It should be noted that a limitation of this study is that it is based on a knockout carried out on a single iPS cell line, so replication in other lines is critical. Nevertheless, our findings strongly support a major role of CHD8 on Wnt/β-catenin signaling, and a connection between *CHD8* and *TCF4*, and several other genes that have been implicated in neuropsychiatric and neurodevelopmental disorders, in particular, members of the *DLX* gene family. In addition, the overlap we detected between the *CHD8* ^*+/−*^ transcriptome and the transcriptomes obtained in idiopathic ASD and BD shows that common molecular pathways exist in different clinical conditions caused by seemingly disparate candidate genes. Identifying such common pathways will facilitate drug discovery in these genetically heterogeneous disorders.

## Conclusions

*CHD8*, which codes for a chromatin-remodeling factor, is mutated in a subgroup of patients with ASD and SZ. RNA-seq analysis of cerebral organoids derived from iPS cells that are heterozygous for a *CHD8* knockout allele, and isogenic controls, shows that CHD8 regulates the expression of other genes implicated in ASD and SZ, notably *TCF4* and *AUTS2*. In addition, extensive overlap was observed for differentially expressed genes (DEGs) found in another study using organoids derived from a family with idiopathic autism, especially for genes involved in GABAergic interneuron development. These findings show molecular convergence of disparate genes involved in the development of ASD and SZ, an observation that will facilitate drug discovery. In addition, pathway analysis of DEGs revealed an enrichment of genes involved in regulating Wnt/β-catenin signaling, a druggable pathway.

## Additional files

Additional file 1: Supplemental methods (DOCX 153 kb)
Additional file 2: Figure S1. (1) Immunohistochemistry (IHS) of control organoid section showing GABA immunoreactivity (GABAergic neurons) in a field of MAP2+ neurons. (2) IHS showing GABA and vGLUT2+ (glutamatergic) neurons. (3). quantitative IHS. CHD8 protein was quantified as described in “Methods” section comparing a control organoid (*CHD8* ^+/+^) with a heterozygous *CHD8* KO organoid (*CHD8* ^+/−^). The images were captured using the same parameters, such as exposure times, for each fluorescence channel was the same for the *CHD8* ^+/+^ and *CHD8* ^+/−^ samples. Images edited in power point using the Picture Tools option were adjusted to the same brightness and contrast levels in *CHD8* ^+/+^and *CHD8* ^+/−^ sections for CHD8 immunoreactivity and for Tuj1 reactivity. (PDF 1507 kb)
Additional file 3: Table S1. Summary of RNA-seq quality and number of differentially expressed genes. (DOCX 14 kb)
Additional file 4: Table S2. old change and level of significance for all genes in RNA-seq analysis: sheet1, results from Kallisto/DESeq2; sheet2, results from TopHat/DESeq2. Log2 fold change is heterozygote/control; pval is uncorrected *p* value; padj is adjusted *p* value for genome-wide significant. Bold type highlights differentially expressed genes at padj < 0.05. NPC binding is based on Sugathan et al. [28]. Log2 fold changes and *p* values from previous findings in NPCs and monolayer neurons are also shown [8]. (XLSX 8994 kb)
Additional file 5: Table S3. Enriched gene ontology (GO) terms for DEGs: Sheet 1, upregulated DEGs; Sheet 2, downregulated DEGs; Sheet 3, IPA canonical pathways. (XLSX 62 kb)
Additional file 6: Table S4. Lists of overlapping DEGs between *CHD8* KO and idiopathic ASD organoids (sheet 1). Gene ontologies for overlapping genes between *CHD8* KO and idiopathic ASD organoids days 11 and 31 (sheets 2 and 3, respectively). (XLSX 58 kb)
Additional file 7: Table S5. List of overlapping DEGs between CHD8 KO organoids, NPCs and neurons, and non-responder and lithium (Li) responder patients with BD (sheet 1). Gene ontologies for overlapping genes between *CHD8* KO and Li non-responders and Li responders (sheets 2 and 3, respectively). (XLSX 22 kb)
Additional file 8: Table S6. Lists DEG from *CHD8* KO organoids, NPCs, and neurons that overlap with ASD and SZ candidate genes (sheet 1). Gene ontologies for overlapping genes between *CHD8* KO and the SZGWAS, AutismKB, and SFARI databases (sheets 2-4, respectively). (XLSX 46 kb)

## Abbreviations

ASD: Autism spectrum disorders
BD: Bipolar disorder
CHD8: Chromodomain helicase DNA-binding protein 8
DEG: Differentially expressed gene
DLX: Distal-less homeobox
ECM: Extracellular matrix
GO: Gene ontology
GWAS: Genome-wide association study
HLA: Human leukocyte antigen
IHC: Immunohistochemistry
IPA: Ingenuity pathway analysis
iPS cell: Induced pluripotent stem cell
KO: Knockout
MHC: Major histocompatibility complex
NPC: Neural progenitor cell
NSC: Neural stem cell
O.C.T: Optimal cutting temperature
OR: Odds ratio
Padj: Adjusted *p* value
PCR: Polymerase chain reaction
Pval: *P* value
qPCR: Quantitative real-time PCR
SZ: Schizophrenia
TPM: Transcripts per kilobase per million reads mapped
WT: Wild type
