# Supplementary material for: CRISPR/Cas9-mediated heterozygous knockout of the autism gene CHD8 and characterization of its transcriptional networks in cerebral organoids derived from iPS cells
Source: Mol Autism. 2017 Mar 20;8:11. doi: 10.1186/s13229-017-0124-1 (PMC5357816; doi:10.1186/s13229-017-0124-1)
Supplement: Additional file 1: — Supplemental methods (DOCX 153 kb) [file 13229_2017_124_MOESM1_ESM.docx]

**Additional file 1: Supplemental methods**

**Subjects used for iPSC development.** The *CHD8*^+/-^ lines were generated in an iPSC line from a control subject who was recruited at the Albert Einstein College of Medicine as part of a larger study to generate iPSCs from control subjects and patients with psychosis who have a 3Mb deletion on 22q11.2. The subject signed a written consent approved by the Albert Einstein College of Medicine IRB (Institutional Review Board). Fibroblasts were obtained from a punch skin biopsy performed by a board-certified dermatologist.

**Harvesting Fibroblasts.** Skin biopsy samples were transferred to a small Petri dish containing 2-3 ml Skin Fibroblast Media (SFM) consisting of RPMI 1640, 10% FBS, 1% pen/strep, 10ng/ml FGF2. The sample was incubated at room temperature for 15 minutes. Medium was carefully aspirated and replaced with 1-2 ml of collagenase type II solution (3mg/ml collagenase II dissolved in DMEM High Glucose [Worthington Biochemical Corp. Lakewood, NJ: GIBCO/Invitrogen, Carlsbad, CA]). The tissue was chopped into small pieces using 2 sterile scalpels, after which they were allowed to incubate at 37^o^C for 1­2 hours depending on size. The sample was then collected in a 15ml falcon tube and washed with SFM (serum free medium). Tissue was collected by centrifugation at 100 x g for 4 minutes. The pelleted sample was then suspended in SFM and plated in a T12.5 ml flask at 37^o^C in 5% CO_2_ for 3 days without changing medium or manipulation, to allow fibroblasts to adhere. Then cells were subsequently fed every 2 days with RPMI 1640 containing 10% FBS until a confluent culture was obtained (~3 weeks).

**Establishing Human iPSCs.**  iPS cell reprogramming was carried out by nucleofection. 1 x 10^6 cells was thawed out and placed in a T75 flask in DMEM/F12 supplemented with 10% FBS and fed every 2 days. Cells were grown to ~50% confluence (~4-5 days), after which they were trypsinized and subjected to nucleofection (~6 x10^5^ cells). Reprogramming was carried out using an Amaxa 4D-Nucleofector (P2 Primary Cell Kit from Lonza cat# V4XP-2012, Program FF-135) with non-integrating plasmids containing OCT4, SOX2, KLF4, L-MYC, LIN28, and a p53 shRNA vector (Addgene Cat. # 27077, 27078, 27080), according to Okita et al., with some modifications [1-3]. iPSCs were maintained on Matrigel plates in mTeSR1 medium (Stem Cell Technologies) with daily feeding in 37^o^C/5% CO_2_/85% humidity.

**Germ line markers, establishing pluripotency by *in vitro* differentiation and karyotype.** Pluripotency for all iPSC lines was confirmed by immunocytochemistry using antibodies (Ab) against Tra-1-60, Tra-1-81, SSEA3 and SSEA4, which are expressed in pluripotent stem cells. In addition, the capacity to differentiate into all 3 germ layers was established by *in vitro* assays, as previously described [2-4]. The markers desmin (mesoderm), α-fetoprotein (endoderm), and βIII-tubulin (ectoderm) were used. A list of the Ab used in the study is shown below. Karyotyping was carried out by Cell Line Genetics (Madison WI). All lines had normal karyotypes**.**

**Immunohistochemistry.** Fixed and embedded cerebral organoids were sectioned (10 µm) in a cryostat at three different intervals. Cells were permeabilized by treating for 1 hour in a buffer mix containing 0.2% Triton-X, 5% donkey serum (DS) in phosphate-buffered saline (PBS). Then, the slice preparations were rinsed with 1% BSA in PBS for 5 minutes. Primary antibodies (Additional file 1: Supplemental methods) were prepared in 1% BSA with appropriate dilutions specific to each antibody and incubated overnight at 4^o^C. After incubation, the slice preparations were washed three times with 1% BSA rinsing solution containing 5% DS (15 minutes for each wash). The samples were incubated in the dark with secondary antibodies conjugated to Alexa fluorophores at 1:300 dilution in 1% BSA in PBS (room temperature for 45 minutes). The samples were then washed three times with rinsing solution, in the dark. The samples were mounted with DAPI-Gold Antifade reagent and visualized after 24 hours.10µm sectioning at day 50 [5-7]**.**

**Quantitative immunohistochemistry.** Cerebral organoid sections (10 microns) were obtained from a CHD8+/+ and CHD8+/- line and labeled with anti-CHD8 and anti-betaIII tubulin (Tuj1) antibodies (see Ab list below), along with DAPI-Gold reagent to visualize nuclei. Slides were visualized under an epifluorescence microscope using 20X magnification. Images were captured using the same parameters, such as exposure times, for each fluorescence channel. Ten random images were captured from each section, which were subsequently analyzed using Fiji software 50 [8]. First, the background signals were removed using the thresholding tool. Then, the pixel intensities were measured, and the mean pixel intensity from each image was recorded, as previously described 50 [9]. The ratios of CHD8 to betaIII tubulin intensities were calculated to represent the CHD8 signal from the neurons only. A 54% decrease in CHD8 immunoreactivity was detected in the CHD8+/- sample (see Additional file 2, Figure S1).

**CRISPR-Cas9 knockout of CHD8**

There are 3 isoforms in the GENCODE annotation, 2 of them contain exon 1 where deletions were designed (see **Figure 1**). The sgRNA sequences were then cloned into the pSpCas9(BB)-2A-Puro (PX459) vector (a gift from Dr. Feng Zhang, Addgene plasmid # 48139). The guide RNAs have the sequences:

sgRNA1 GTCATCGAACAGATCCATGATGG

sgRNA2 GACTGATGACAGCTTTAACCAGG

Regarding the choice of exon 1 as target, this region is mutated in ASD and is expressed in the brain, as well as human NPCs and neurons. To confirm expression in organoids, we mapped reads by TopHat, and calculated RPKM values for exon 1 and constitutive exons, respectively (see **Table 1**). From the ratio of RPKM between exon1 and constitutive exons, we could estimate that in WT samples ~75% of CHD8 transcripts contain exon 1, suggesting that ENST00000430710, which bypasses exon 1 is the minor isoform.

**Figure 1: Gene annotation of CHD8 in GENCODE**

**Table 1: RPKM values of CHD8**

|  | **Reads aligned to constitutive exons** | **Reads aligned to exon1** | **RPKM(exon1) /**  **RPKM(constitutive exons)** |
| --- | --- | --- | --- |
| **CHD8+/+ A** | **1,675** | **192** | **0.8011** |
| **CHD8+/+ B** | **1,433** | **145** | **0.7072** |
| **CHD8+/- A** | **2,269** | **219** | **0.6746** |
| **CHD8+/- B** | **2,393** | **207** | **0.6046** |
| **CHD8+/- C** | **2,172** | **208** | **0.6693** |
| **CHD8+/- D** | **677** | **65** | **0.6710** |

iPS cells were cultured and fed daily in mTeSR1 (Stem Cell technologies) on Matrigel (BD) coated plates at 37^o^C in a humidified incubator in 5% CO2. Cells were maintained in log phase growth and differentiated cells were manually removed. iPS cells were exposed to 10uM ROCK Inhibitor for ~4 hours to improve cell survival during nucleofection. After 4 hours, growth medium was aspirated and the cells were rinsed with DMEM/F12. iPS cells were dissociated into single cells using accutase and harvested. Nucleofection was performed using the Amaxa-4D Nucleofector Basic Protocol for Human Stem Cells (Lonza) according to the manufacturer’s instructions. Briefly, 8x10^5 cells and 5ug of the CRISPR/Cas9 plasmids with either sgRNA1 or sgRNA2 were nucleofected using the P3 Primary Cell 4D-Nucleofector X Kit L with program CA-137. Cells were resuspended in mTeSR1 + 10uM ROCK Inhibitor and placed in one well of a 6-well Matrigel-coated plate. The following day, cells were fed with fresh mTeSR1, and were subsequently fed with fresh medium every day. Initially on days 4-6, cells were exposed to 0.5ug/ml puromycin for 6 hours. Afterwards they were exposed for 24 hours until day 14. Puromycin-resistant colonies were picked and expanded in mTeSR1 without further puromycin treatment.

“TA” cloning was used to identify the knockout alleles. A 479 bp PCR amplicon flanking the CRSPR/cas9 targeted sites was generated using the primers 5’-CTGTAAGACAGGTTGGGCTG-3’ and 5’-CTTGTTTCTTGCCTCTATACTTGA-3’. The PCR product was purified and ligated into pCR™2.1 using a TA Cloning Kit developed by Life Technologies following the manufacturer’s protocol. Recombinant plasmids were introduced into competent E. coli and selected in ampicillin. Plasmid DNA was extracted and sequenced across the insert using one of the PCR primers.

The sgRNA1 KO results in the loss of an NlaIII restriction site, while the sgRNA2 KO results in the loss of an MseI restriction site. *CHD8*^+/-^ clones A,B and D are derived from sgRNA2 and are heterozygous for a 10 bp deletion that leads to a frameshift mutations and premature stop codon in exon 1, as previously described [10]. The 10 bp deletion begins at position chr14:21899722 (hg19). *CHD8*^+/-^ clone C is derived from sgRNA1 and has a 2 bp deletion in exon 1; it also leading to a premature stop codon in exon 1. The 2 bp deletion begins at chr14:21899785. *CHD8*^-/-^ is derived from sgRNA2; it is homozygous for a single base deletion (T) at position chr14:21899726.

Western blotting was used to confirm that the CHD8^+/-^ lines expressed lower levels of CHD8 protein in NPCs, while homozygous KO, *CHD8*^-/-^ did not express it at all [10]. Specifically, cell lysates from NPCs differentiated from wild type and KO iPSCs were separated by SDS PAGE, transferred to PVDF membranes, and then blotted with anti-CHD8 antibody (Bethyl Cat #A301-224A). Anti-actin antibody (BD Biosciences, Cat # 612656) was used for loading control.

**Reverse transcribed PCR (RT-PCR) and quantitative real-time PCR (qPCR)**

Total RNA was extracted using a miRNeasy Kit according to the manufacturer’s instructions (Qiagen). An additional treatment with DNase1 (Qiagen, Valencia, CA) was included to remove genomic DNA. Reverse transcribed PCR (RT-PCR) was performed using a OneStep RT-PCR Kit (Qiagen, Valencia, CA) according to the manufacturer’s instructions for semi-quantitative analysis. Biorad’s iScript cDNA Synthesis Kit was used to generate cDNA as a template for quantitative PCR (qPCR), which was carried out using the ABI 7900HT Real-Time PCR System (Applied Biosystems, Foster City, CA). Each reaction consisted of cDNA, primers, and Power SYBR Green PCR Master Mix (Applied Biosystems, Foster City, CA) in an 8 μl volume. Melting curve analysis of target sequences showed that all primers used in this study generated amplicons that had a single peak, without primer-dimer artifacts. Primer concentrations were optimized prior to use in qPCR experiments. Relative changes in gene expression were calculated using the 2^-∆∆Ct^ method with β2-microglobulin (β2M) as a reference gene described [2, 4, 8]. Each qPCR was carried out in triplicate, with each triplicate data point repeated 3 times. For the triplicates, only samples that differed by <0.3 Ct values were used in the final calculations. Less than ~5% of samples fell out of this range. In addition, standard curves were generated for each gene using a 50-fold dilution range. Relative changes in gene expression were calculated using the 2^-∆∆Ct^ method with β2-microglobulin (β2M) as a reference gene. Significant differences in gene expression were assessed using a two-tailed student’s T-test.

**Nonsense mediate decay of KO allele**

RNA reads across the 2 bp and 10 bp deletions were assessed in our RNA-seq data. As shown in **Table 2** below, there were fewer reads across these regions in the CHD8^+/-^ KO samples compared to the WT isogenic controls (A, B and C are the organoids with the 10 bp deletion; D contains the 2 bp deletion). This is highly suggestive of nonsense mediated decay (NMD).

**Table 2: read counts across the deleted regions**

|  | WT organoids | | KO organoids | | | |
| --- | --- | --- | --- | --- | --- | --- |
| Read counts | +/+ A | +/+ B | +/- A | +/- B | +/- C | +/- D |
| WT allele | 22 | 25 | 34 | 33 | 14 | 37 |
| KO allele | 0 | 0 | 8 | 1 | 1 | 3 |
| Total Mapped RNA-seq reads  (reference for cross-sample comparison) | 33,724,530 | 33,014,732 | 46,975,256 | 51,229,638 | 31,118,330 | 47,091,810 |

Although NMD is occurring, total CHD8 RNA is only reduced by ~12% in the CHD8^+/-^ samples (**Table 3**). This suggests that CHD8 mRNA and CHD8 protein levels are not coupled. This is not especially unusual since there is poor correlation between mRNAs and corresponding translated proteins for many genes. Uncoupling can occur at a number of levels, including translation initiation, translation elongation, choice of open reading frame, folding of the emerging protein, cis-acting genetic factors, and variations in the global pools of translation components (ribosomes, tRNAs, mRNAs, and translation factors) [11-15]. However, the mechanism underlying CHD8 mRNA and protein uncoupling is not known at this time.

A similar uncoupling was observed in a CHD8^-/-^ line we obtained in our CRISPR/Cas9 knockout experiment [10].

**Table 3: DESeq2 normalized reads for CHD8**

| Normalized read counts: CHD8 | | | | | | log2FoldChange | pvalue | padj |
| --- | --- | --- | --- | --- | --- | --- | --- | --- |
| WT Organoids | | KO Organoids | | | | -0.177 | 0.265 | 0.636 |
| ++ A | ++ B | +/- A | +/- B | +/- C | +/- D |  |  |  |
| 2307 | 1852 | 1840 | 2009 | 1717 | 1729 |  |  |  |

**PCR primers used in this study**

**Characterizing iPSCs**

**Gene Forward Reverse**

OCT4 plasmid CATTCAAACTGAGGTAAGGG TAGCGTAAAAGGAGCAACATAG

KLF4 plasmid CCACCTCGCCTTACACATGAAGA GCGTAAAAGGAGCAACATAG

SOX2 plasmid TTCACATGTCCCAGCACTACCAGA TTGTTTGACAGGAGCGACGAT

L-MYC plasmid GGCTGAGAAGAGGATGGCTAC TTTGTTTGACAGGAGCGACGAT

LIN28 plasmid AGCCATATGGTAGCCTCATGTCCGC TAGCGTAAAAGGAGCAACATAG

**Primers used to amplify region containing the CRISPR-engineered region**

CHD8KO-A TGGCAGGCTGAGTGGTATAA GAGTAGTCCTCTTACATTGTGTA

CHD8KO-B CTGTAAGACAGGTTGGGCTG CTTGTTTCTTGCCTCTATACTTGA

**Primers used for qPCR**

**Gene Forward Reverse**

β2M GCTCGCGCTACTCTCTCTTT CAATGTCGGATGGATGAAAC

SOX2 CAACCAGAAAAACAGCCCGG TGTGCATCTTGGGGTTCTCC

TCF4 GCCACCTGTCTCCTTACCAA TGGGGTAGAAGGGATGTTTG

PAX6 AACAGACACAGCCCTCACAAACA CGGGAACTTGAACTGGAACTGAC

CNTNAP2 GGATGCTCTACAGCGACACA TCTCCATTCCAATCCAGAGG

HMGA2 GGTGCCACCCACTACTCTGT TGAGATTGAAAGTGCCTTGG

RELN AGCCCTTCTCAAGACCGAGT TCACGGACACATCAACATGA

MEG3 GGCCTCCCCTTGAGTAGAGA CAACAGCCCTGTGAGGTAGG

DLX6-AS1 AGGAAAACCCACTCCCTTGG TGATGATGGTGTCCAGGAGC

MIAT TGTCTCCATTTGCTCAGTGC TCAGGATGGTGCACTCTCAG

CRNDE GATGACTCATTGTGAGTGCTAG CCATGTTCTTTGCATCTAGATT

**Antibodies used in this study**

| Antibody | Company | Catalog # | dilution |
| --- | --- | --- | --- |
| Anti-human Tra 1-60 | eBioscience | 12-8863-80 | 1:100 |
| Anti-human Tra 1-81 | eBioscience | 12-8883-80 | 1:100 |
| AF488 Anti-mouse/human SSEA-3 | eBioscience | 53-8833-71 | 1:100 |
| AF488 Mouse anti SSEA-4 | BD Pharmingen | 560308 | 1:100 |
| Anti-Tubulin, beta III isoform | Millipore | MAB1637 | 1:500-1:100 |
| Desmin Ab-1 | ThermoScientific | MS-376-S | 1:100 |
| Anti-human/mouseα-Fetoprotein | R & D | MAB1368 | 1:100 |
|  |  |  |  |
|  |  |  |  |
| Anti-GAD65/67 | Sigma | G5163 | 1:1000 |
| Ms anti- Vglut2 | Millipore | MAB5504 | 1:100-1:50 |
| Rabbit neuronal class III β-tubulin | Fisher | NC9168644 | 1:1000 |
| Mouse Anti-Reelin | Millipore | MAB5364 | 1:500 |
| Rabbit Anti-Vglut2 | SYSY | 135403 | 1:1000 |
| Rabbit Anti-SOX2 Antibody | STEMCELL | 60055.1 | 1:100-1:50 |
| Rabbit Anti-GABA | SIGMA-ALDRICH | A2052 | 1:1000 |
| Anti-NeuN Antibody | abcam | ERP12763 | 1:300 |
| Rabbit CHD8 Antibody | BETHYL | A301-224A | 1:250-1:1000 |
| Rabbit Pax-6 Antibody | COVANCE | PRB-278P | 1:300 |
| Mouse Anti-Vimentin | invitrogen | 18-0052 | 1:100 |

References

1. Okita K, Matsumura Y, Sato Y, Okada A, Morizane A, Okamoto S, Hong H, Nakagawa M, Tanabe K, Tezuka K, Shibata T, Kunisada T, Takahashi M, Takahashi J, Saji H, Yamanaka S: **A more efficient method to generate integration-free human iPS cells.** Nat Methods 2011, **8**(5):409-412.

2. Chen J, Lin M, Foxe JJ, Pedrosa E, Hrabovsky A, Carroll R, Zheng D, Lachman HM: **Transcriptome Comparison of Human Neurons Generated Using Induced Pluripotent Stem Cells Derived from Dental Pulp and Skin Fibroblasts.** PLoS One 2013, **8**(10):e75682.

3. Pedrosa E, Sandler V, Shah A, Carroll R, Chang C, Rockowitz S, Guo X, Zheng D, Lachman HM: **Development of Patient-Specific Neurons in Schizophrenia Using Induced Pluripotent Stem Cells.** J Neurogenet 2011, .

4. Chen J, Lin M, Hrabovsky A, Pedrosa E, Dean J, Jain S, Zheng D, Lachman HM: **ZNF804A Transcriptional Networks in Differentiating Neurons Derived from Induced Pluripotent Stem Cells of Human Origin.** PLoS One 2015, **10**(4):e0124597.

5. Fatemi SH, Stary JM, Earle JA, Araghi-Niknam M, Eagan E: **GABAergic dysfunction in schizophrenia and mood disorders as reflected by decreased levels of glutamic acid decarboxylase 65 and 67 kDa and Reelin proteins in cerebellum.** Schizophr Res 2005, **72**(2-3):109-122.

6. Fatemi SH, Folsom TD: **The neurodevelopmental hypothesis of schizophrenia, revisited.** Schizophr Bull 2009, **35**(3):528-548.

7. Hashemi E, Ariza J, Rogers H, Noctor SC, Martinez-Cerdeno V: **The Number of Parvalbumin-Expressing Interneurons Is Decreased in the Medial Prefrontal Cortex in Autism.** Cereb Cortex 2016.

8. Jensen EC: **Quantitative analysis of histological staining and fluorescence using ImageJ.** Anat Rec (Hoboken) 2013, **296**(3):378-381.

9. Schindelin J, Arganda-Carreras I, Frise E, Kaynig V, Longair M, Pietzsch T, Preibisch S, Rueden C, Saalfeld S, Schmid B, Tinevez JY, White DJ, Hartenstein V, Eliceiri K, Tomancak P, Cardona A: **Fiji: an open-source platform for biological-image analysis.** Nat Methods 2012, **9**(7):676-682.

10. Wang P, Lin M, Pedrosa E, Hrabovsky A, Zhang Z, Guo W, Lachman HM, Zheng D: **CRISPR/Cas9-mediated heterozygous knockout of the autism gene CHD8 and characterization of its transcriptional networks in neurodevelopment.** Mol Autism 2015, **6**:55-015-0048-6. eCollection 2015.

11. Maier T, Guell M, Serrano L: **Correlation of mRNA and protein in complex biological samples.** FEBS Lett 2009, **583**(24):3966-3973.

12. Cenik C, Cenik ES, Byeon GW, Grubert F, Candille SI, Spacek D, Alsallakh B, Tilgner H, Araya CL, Tang H, Ricci E, Snyder MP: **Integrative analysis of RNA, translation, and protein levels reveals distinct regulatory variation across humans.** Genome Res 2015, **25**(11):1610-1621.

13. Vogel C, Marcotte EM: **Insights into the regulation of protein abundance from proteomic and transcriptomic analyses.** Nat Rev Genet 2012, **13**(4):227-232.

14. Rodnina MV: **The ribosome in action: Tuning of translational efficiency and protein folding.** Protein Sci 2016, **25**(8):1390-1406.

15. Ly T, Ahmad Y, Shlien A, Soroka D, Mills A, Emanuele MJ, Stratton MR, Lamond AI: **A proteomic chronology of gene expression through the cell cycle in human myeloid leukemia cells.** Elife 2014, **3**:e01630.
