# Supplementary figures and images for: CRISPR/Cas9-mediated heterozygous knockout of the autism gene CHD8 and characterization of its transcriptional networks in cerebral organoids derived from iPS cells
Source: Mol Autism. 2017 Mar 20;8:11. doi: 10.1186/s13229-017-0124-1 (PMC5357816; doi:10.1186/s13229-017-0124-1)

A. GABA/MAP2/DAPI

GABA

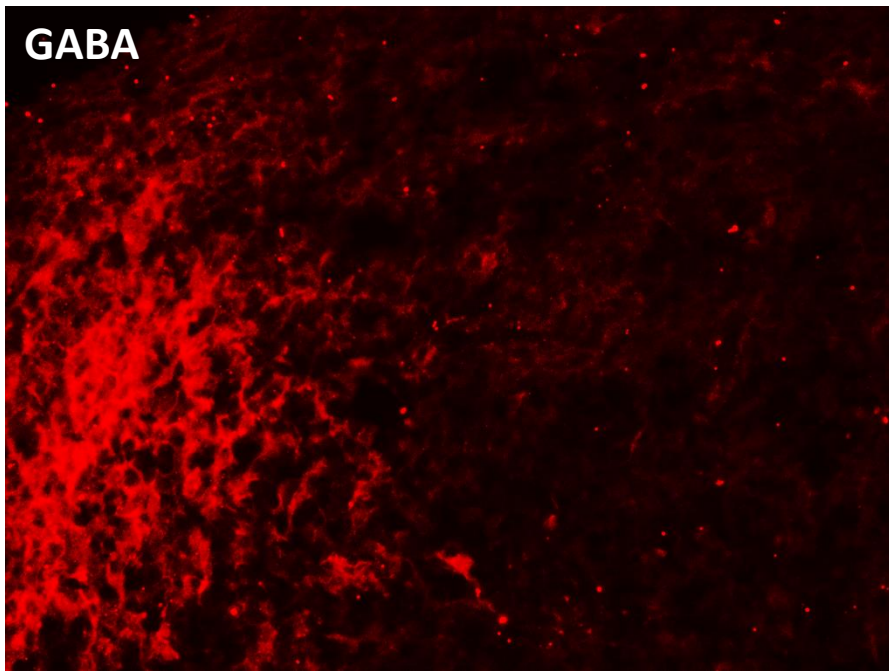

MAP2

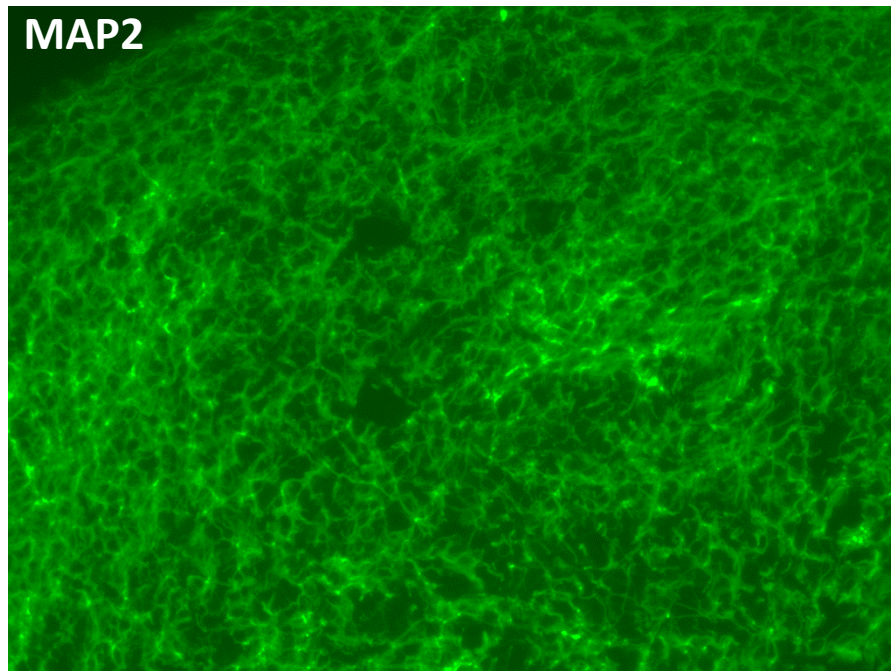

DAPI

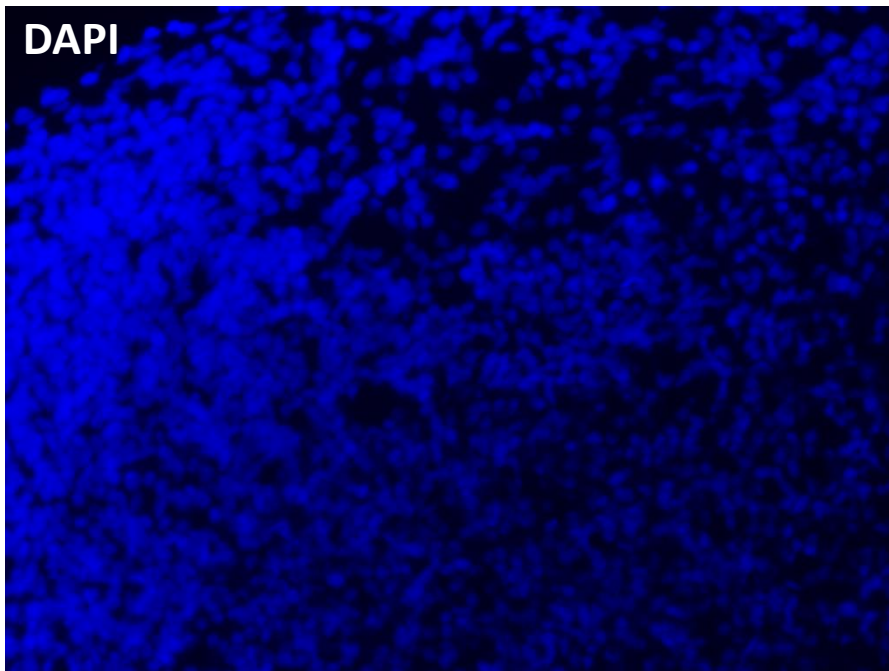

MERGED

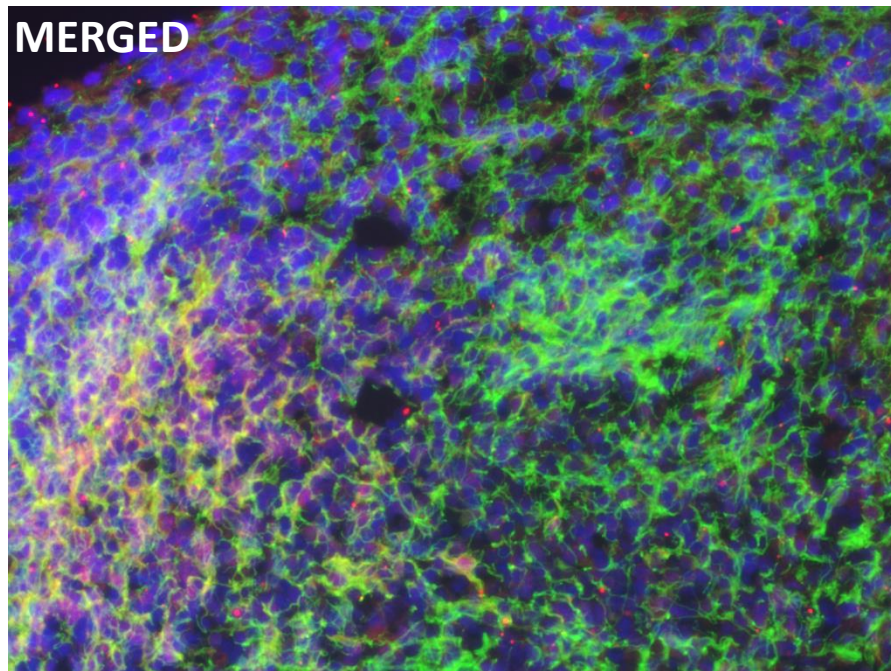

**B. GABA/vGLUT/DAPI**

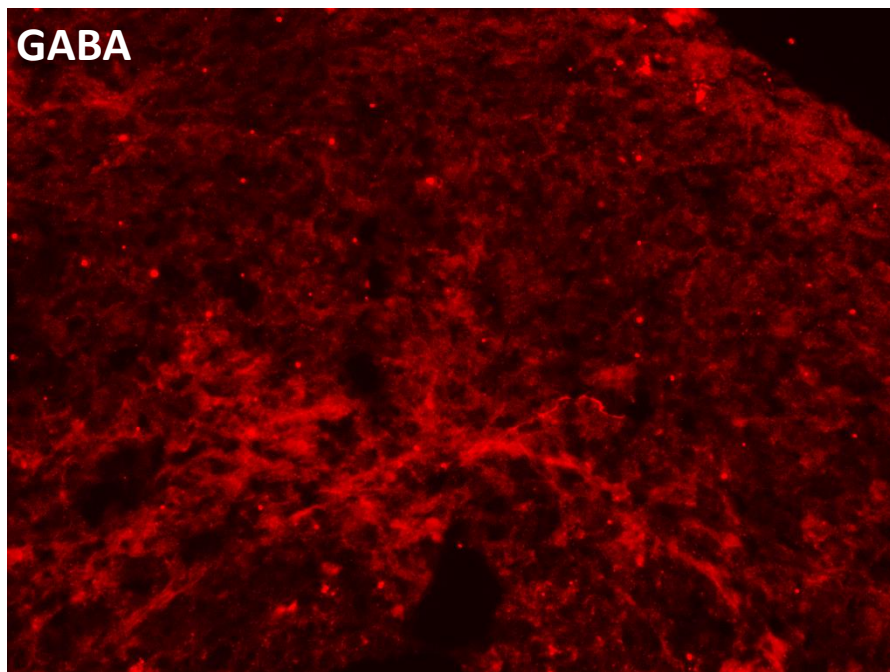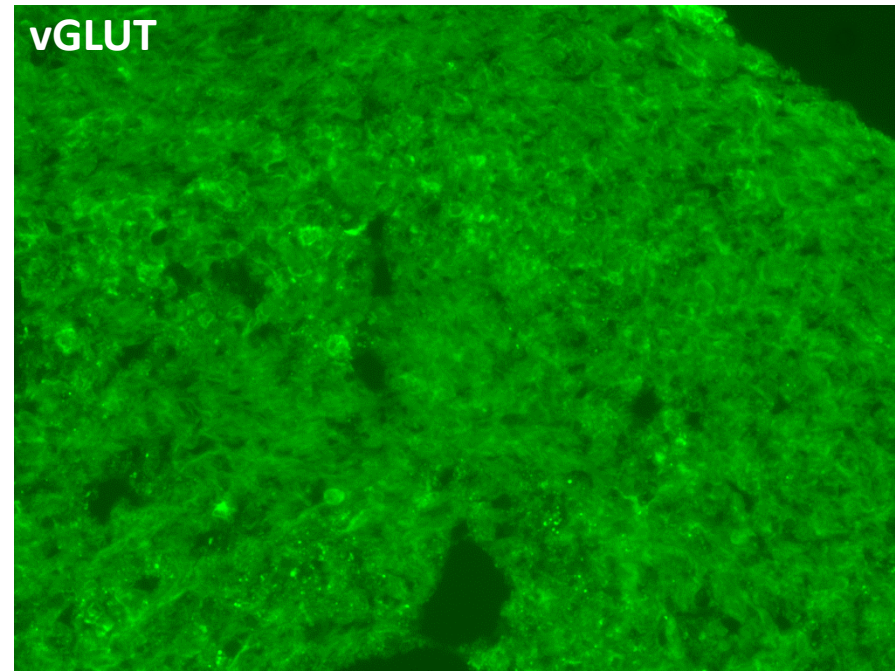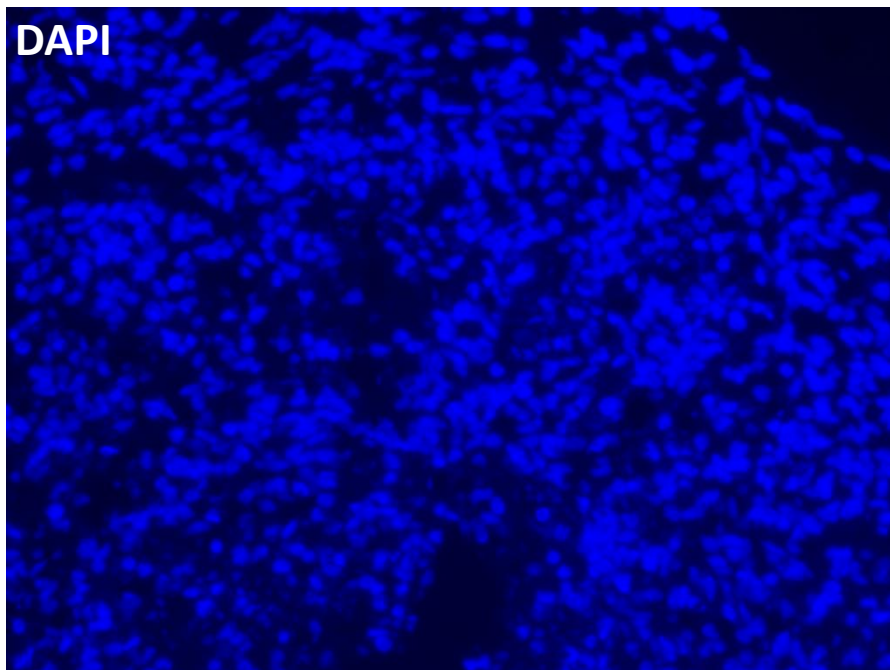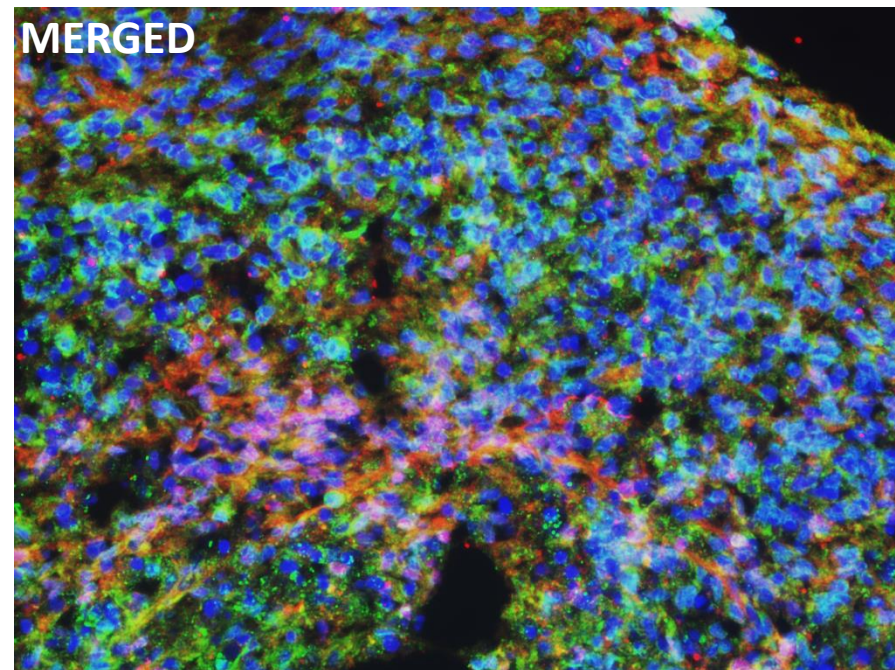

C. CHD8/Tuj1

Control

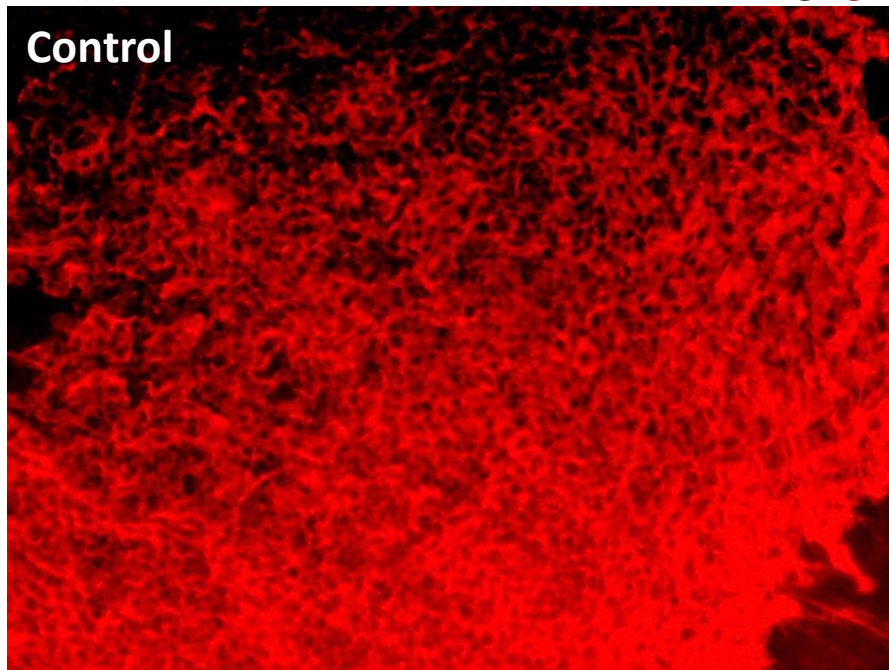

Control

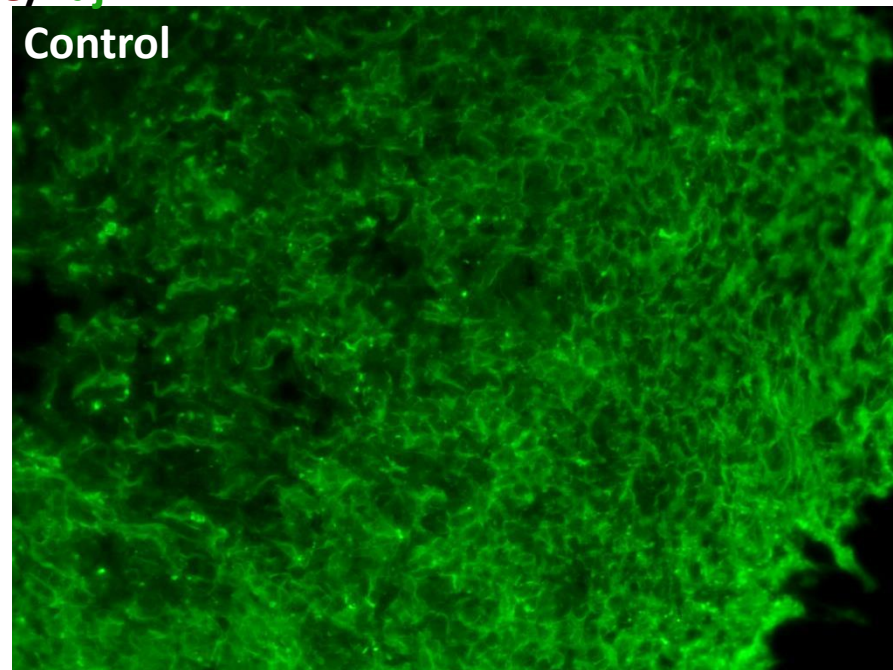

CHD8+/-

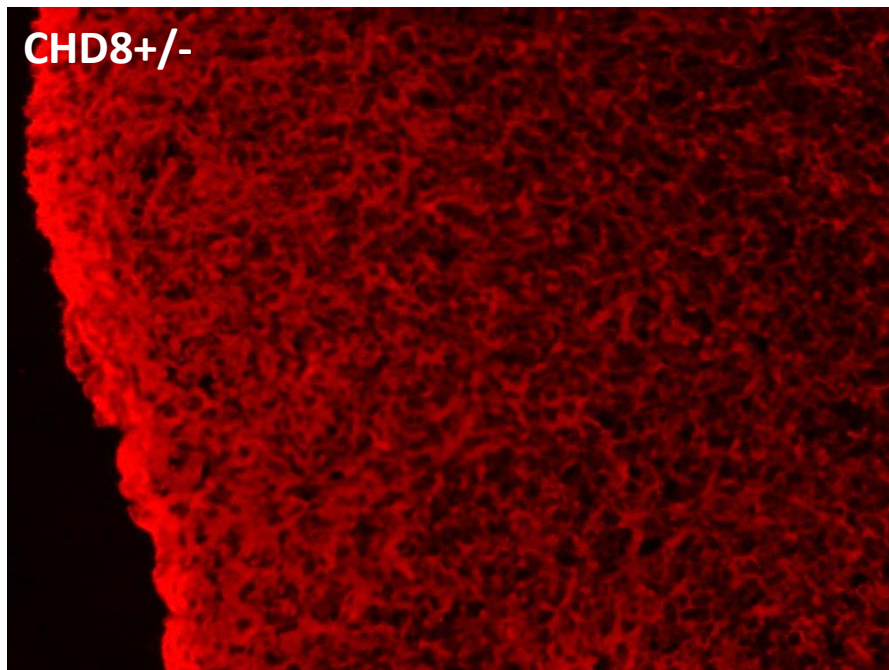

CHD8+/-

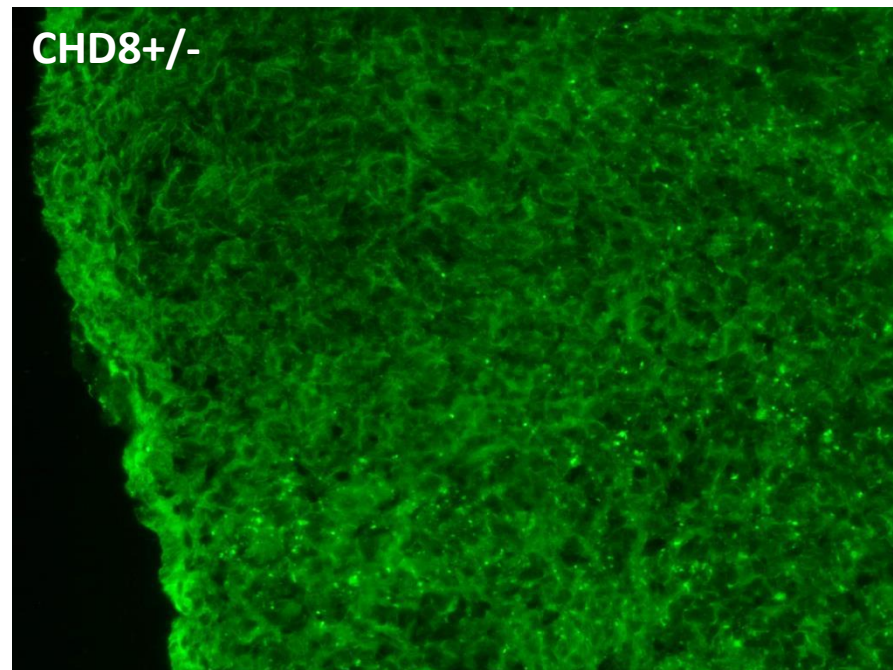

Supplement: Additional file 2: Figure S1. — (1) Immunohistochemistry (IHS) of control organoid section showing GABA immunoreactivity (GABAergic neurons) in a field of MAP2+ neurons. (2) IHS showing GABA and vGLUT2+ (glutamatergic) neurons. (3). quantitative IHS. CHD8 protein was quantified as described in “Methods” section comparing a control organoid (CHD8 +/+) with a heterozygous CHD8 KO organoid (CHD8 +/−). The images were captured using the same parameters, such as exposure times, for each fluorescence channel was the same for the CHD8 +/+ and CHD8 +/− samples. Images edited in power point using the Picture Tools option were adjusted to the same brightness and contrast levels in CHD8 +/+and CHD8 +/− sections for CHD8 immunoreactivity and for Tuj1 reactivity. (PDF 1507 kb) [file 13229_2017_124_MOESM2_ESM.pdf]
