# Supplementary material for: CRISPR/Cas9-mediated heterozygous knockout of the autism gene CHD8 and characterization of its transcriptional networks in cerebral organoids derived from iPS cells
Source: Mol Autism. 2017 Mar 20;8:11. doi: 10.1186/s13229-017-0124-1 (PMC5357816; doi:10.1186/s13229-017-0124-1)
Supplement: Additional file 3: Table S1. — Summary of RNA-seq quality and number of differentially expressed genes. (DOCX 14 kb) [file 13229_2017_124_MOESM3_ESM.docx]

**Additional file 2: Table S1. RNA-seq reads and number of differentially expressed genes (DEGs)**

| **sample** | **trimmed pairs** |
| --- | --- |
| CHD8^+/+^ A | 18,875,274 |
| CHD8^+/+^ B | 20,345,793 |
| CHD8^+/-^ A | 26,980,058 |
| CHD8^+/-^ B | 30,785,992 |
| CHD8^+/-^ C | 17,655,010 |
| CHD8^+/-^ D | 26,895,260 |

| DEGs (FDR<0.05) | Het_wt |
| --- | --- |
| 🡹 | 288 |
| 🡻 | 271 |

CHD8^+/+^ is the parent line (wild type; wt); het (heterozygote) is CHD8^+/-^
